# Identification and Analysis of Potential Genes Regulated by an Alphasatellite (TYLCCNA) that Contribute to Host Resistance against Tomato Yellow Leaf Curl China Virus and Its Betasatellite (TYLCCNV/TYLCCNB) Infection in *Nicotiana benthamiana*

**DOI:** 10.3390/v11050442

**Published:** 2019-05-15

**Authors:** Chaohu Luo, Zhan Qi Wang, Xianan Liu, Liling Zhao, Xueping Zhou, Yan Xie

**Affiliations:** 1 State Key Laboratory of Rice Biology, Institute of Biotechnology, Zhejiang University, Hangzhou 310058, China; teapotmrluo@163.com (C.L.); 18753363127@163.com (X.L.); zhaolilingyunnan@163.com (L.Z.); zzhou@zju.edu.cn (X.Z.); 2 Key Laboratory of Vector Biology and Pathogen Control of Zhejiang Province, College of Life Sciences, Huzhou University, Huzhou 313000, China; zhqwang@zju.edu.cn; 3 Key Laboratory for Biology of Plant Diseases and Insect Pests, Institute of Plant Protection, Chinese Academy of Agricultural Sciences, Beijing 100193, China

**Keywords:** *Nicotiana benthamiana*, TYLCCNV/TYLCCNB, TYLCCNA, transcriptomics, VIGS, host resistance

## Abstract

Recently, begomovirus/betasatellite disease complexes were found to be associated with alphasatellites, and their presence modulated disease symptoms and/or viral DNA accumulation in infected plants. However, the biological functions of alphasatellites during begomovirus/betasatellite infections remain unclear. Tomato yellow leaf curl China virus (TYLCCNV) associated with a betasatellite (TYLCCNB) is a widespread monopartite begomovirus in China. In the Yunnan province of China, the TYLCCNV/TYLCCNB disease complex is found in association with an alphasatellite (TYLCCNA). In this study, in order to explain the mechanisms underlying TYLCCNV/TYLCCNB infection and reductions in viral DNA accumulation caused by TYLCCNA, we analyzed the transcriptome profiles of *Nicotiana benthamiana* seedlings challenged by TYLCCNV/TYLCCNB or TYLCCNV/TYLCCNB/TYLCCNA using RNA sequencing. In total, 2272 and 1207 differentially expressed genes (DEGs) were identified to respond to TYLCCNV/TYLCCNB and TYLCCNV/TYLCCNB/TYLCCNA infections, respectively. Compared with the DEGs in the TYLCCNV/TYLCCNB-infected *N. benthamiana* seedlings, the number of DEGs in plants co-infected with TYLCCNV/TYLCCNB + TYLCCNA was significantly reduced. Additionally, 36 DEGs were identified to be regulated by TYLCCNA, six of which were further analyzed using the virus-induced gene silencing (VIGS) approach. Silencing of these six TYLCCNA responsive DEGs caused more severe disease symptoms and higher viral DNA accumulation levels, suggesting that TYLCCNA responsive DEGs may attenuate TYLCCNV/TYLCCNB infection.

## 1. Introduction

Viruses in the family *Geminiviridae* are circular single-stranded DNA phytopathogens packaged in viral twin particles [1,2,3,4]. They are currently divided into nine genera: *Begomovirus*, *Mastrevirus*, *Topocuvirus*, *Curtovirus*, *Becurtovirus*, *Turncurtovirus*, *Eragrovirus*, *Grablovirus* and *Capulavirus* by the International Committee on Taxonomy of Viruses [5]. The genus *Begomovirus*, members of which are known as whitefly-transmitted geminiviruses (WTGs) [6], contains more than 350 species or members, and is the largest genus within the *Geminiviridae* [7]. The prevalence and severity of begomovirus diseases has increased significantly in the past two decades [8,9,10], greatly affecting agriculture and causing considerable losses in infected fields [11,12]. Begomoviruses are classified as New World (the Americas) or Old World (Europe, Africa, Asia and Australasia) based on their geographic origins [6]. The genomes of begomoviruses in the New World are bipartite, whereas the Old World begomoviruses have either a monopartite or bipartite genome [13].

Monopartite begomoviruses are usually found in association with two types of satellite molecules that are referred to as betasatellites and alphasatellites [4,13]. Betasatellites are circular single-stranded DNA molecules that are half the size of those of their helper viruses and have a conserved sequence (satellite conserved region, SCR) and encode a protein (known as βC1) in the complementary-sense orientation [14]. βC1, which is a pathogenicity determinant, has functions as an RNA silencing suppressor and a symptom determinant [15,16,17]. The βC1 is also implicated in the cell-to-cell movement of viruses instead of the function of movement protein (MP) [18]. In addition, the βC1 also can interact with ASYMMETRIC LEAVES 1 (AS1) to suppress jasmonic acid (JA)-mediated plant defenses against the whitefly [19,20].

Alphasatellites are frequently found to be associated with begomovirus/betasatellite disease complexes [21,22,23,24]. They are circular single-stranded DNA molecules, which have approximately 1375 nucleotides and possess a highly conserved genome organization. In the virus-sense orientation, alphasatellites encode a replication initiator protein (alpha-Rep) and thereby are able to self-replicate, but depend on their helper viruses for encapsidation and movement in plants, as well as for insect transmission [13]. The precise impact of alphasatellites on begomovirus or begomovirus/betasatellite infections remains unclear [13,23,25]. However, some alphasatellites have been shown to modulate viral symptoms and/or reduce or increase the accumulation of their helper virus and/or betasatellite DNA in the early stages of infections [26,27,28,29].

In China, alphasatellites are frequently reported to infect crops and weeds with begomovirus/betasatellite complexes, and these alphasatellites are classified as one of three types according to their phylogenetic relationships [22,30,31]. Among these three, type I, which is in association with the tomato yellow leaf curl China virus (TYLCCNV)/betasatelite (TYLCCNB) complex, has the largest number of alphasatellites [22]. At present, research on alphasatellites is mainly focused on their molecular characterization; however, little is known about their biological functions during begomovirus/betasatellite infections. In this study, we investigated the biological functions of widespread alphasatellite (TYLCCNA) during TYLCCNV/TYLCCNB infection in China using Illumina RNA sequencing (RNA-seq) technology coupled with a virus-induced gene silencing (VIGS)-based assay. This study allowed us to identify and analyze potential host genes related to the alphasatellite TYLCCNA in *N. benthamiana*. Although the functions of these differentially expressed genes (DEGs) still require examination, these results enhance our understanding of the functions of alphasatellites during begomovirus/betasatellite infections.

## 2. Materials and Methods

### 2.1. Plant Materials, Growth Conditions and Agroinoculations

*N. benthamiana* plants were grown in a greenhouse at a constant temperature of 25 °C with supplementary lighting corresponding to a 16/8-h day/night schedule as described in previous research [32]. Infectious clones of TYLCCNV, TYLCCNB and TYLCCNA were constructed as described by Zhou et al. [33]. Agroinoculations with five different groups of infectious clones: TYLCCNV, TYLCCNV/TYLCCNA, TYLCCNV/TYLCCNB, TYLCCNV/TYLCCNB/TYLCCNA and pBINPLUS empty vector (mock control) were performed as described in previous research [32]. For the agroinoculation of *N. benthamiana*, seedlings at the 5- to 6-leaf stage were infiltrated with the *Agrobacterium tumefaciens* mixtures which contained equal volumes of the separate bacterial cultures for each experiment. Plants agroinfiltrated with the clone of pBINPLUS were used as mock controls.

### 2.2. Genomic DNA Extraction and Southern Blot Analysis

Total genomic DNA was extracted from systemically infected leaves of *N. benthamiana* seedlings at 7 days post-inoculation (dpi) using the cetyltrimethylammonium bromide (CTAB) method as described in previous research [4]. Nucleic acids were fractionated on a 1.5% agarose gel in Tris-borate-EDTA (TBE) buffer (90 mM Trisborate, 90 mM H_3_BO_3_, 2 mM EDTA, pH 8.0) and transferred to Hybond-N^+^ membranes (Amersham Biosciences, Piscataway, NJ, USA). Subsequently, these nucleic acids were hybridized with digoxin-labeled probes using a DIG High Prime DNA Labeling and Detection Starter Kit II (Roche Diagnostics, Mannheim, Germany). The quantitative assessment was processed by Quantity One Software (http://www.bio-rad.com) as described in previous research [34].

### 2.3. Transcriptome Sequencing and Unigene Annotation

RNA-Seq libraries were constructed, sequenced and assembled as described in previous research [35]. In brief, 15 strand-specific barcoded RNA-sequencing libraries constructed from mock- and virus-infected *N. benthamiana* plants at 7 dpi were pooled and sequenced using an Illumina HiSeq 2000 system (Illumina, San Diago, CA, USA) with a paired-end mode. Adaptor and low-quality sequences were removed from the raw reads and the resulting clean reads were then combined with overlapping nucleic acid sequences using Trinity software [36] to form contigs or unigenes as described in previous research [37,38].

The unigenes were annotated using BLASTx to several protein databases including NCBI (http://www.ncbi.nlm.nih.gov/), Swiss-Prot protein (http://www.uniprot.org/), Kyoto Encyclopedia of Genes and Genomes (KEGG) (http://www.genome.jp/kegg/) and COG database (https://www.ncbi.nlm.nih.gov/COG/) with an E-value threshold of 1E−5 as described in previous research [39]. A classification of the metabolic pathway(s) that the annotated genes are involved in was produced with Gene Ontology (GO) and KEGG classifications by Blast2GO (https://www.blast2go.com/) as described by Shen et al. [40].

### 2.4. Differential Gene Expression, GO and KEGG Enrichment Analyses

For differential expression analysis, the number of mapped reads for each pairwise comparison was counted and normalized into an expected fragments per kilobase of transcript per million fragments sequenced (FPKM) value as described in previous research [41]. To determine significant differences, an absolute value of log2(Ratio) ≥ 1 and a false discovery rate (FDR) ≤ 0.05 were used as thresholds as described in previous research [42,43]. GO and KEGG enrichment analyses of the DEGs between different pairwise comparisons were performed and the FDR-corrected *p*-values (*Q*-values) ≤ 0.05 were considered to be differentially enriched as described in previous research [44].

### 2.5. Reverse Transcription Quantitative Real-Time PCR (RT-qPCR) Analysis

Total RNA from different leaf samples was isolated using the RNAiso Plus Kit (TaKaRa, Dalian, China) and genomic DNA contamination was cleaned by DNase I. cDNA was synthesized using ReverTra Ace RT-qPCR RT Master Mix (Toyobo, Osaka, Japan) according to the manufacturer′s instructions. RT-qPCR was performed using the SYBR Premix Ex Taq Kit (TaKaRa, Dalian, China) and the PCR amplification conditions were as follows: 95 °C for 1 min; 45 cycles of 95 °C for 10 s, 56 °C for 15 s and 72 °C for 20 s. For each candidate gene, the PCR reactions were performed in triplicate, and expression data were normalized with the expression level of *NbACTIN2* as described in previous research [4]. The primers used for the RT-qPCR analysis are provided in the Supplementary Table S1.

### 2.6. Virus-Induced Gene Silencing (VIGS) Assay

In our study, the tobacco rattle virus (TRV)-based VIGS system [45] was employed to silence the *N. benthamiana flavonoid biosynthesis oxidoreductase protein* (*FBOP*; CL3311.Contig2_All), *N. benthamiana β-1,3-glucanase* (*β-1,3-G*; CL16016.Contig3_All), *N. benthamiana glutathione S-transferase-like* (*GSTL*; Unigene47881_All), *N. benthamiana lectin-domain receptor-like kinase* (*LecRLK*; CL8323.Contig1_All), *N. benthamiana UDP-arabinose 4-epimerase-like* (*UA4EL*; Unigene72008_All) and *N. benthamiana arogenate dehydrogenase 2* (*AD2*; Unigene40989_All) genes. VIGS constructs were generated in accordance with the method as described by Zhong et al. [4] at the *Kpn*I/*Bam*HI restriction enzyme sites of the pTRV2 plasmid and the primers used in the plasmid constructs are provided in the Supplementary Table S1. The agrobacterium-mediated inoculation of *N. benthamiana* was carried out as described in previous research [4], and agroinfiltration with pTRV1 and pTRV2:GFP was used as the control. For each construct, twenty-four individual plants were used in the VIGS experiment. Ten days after inoculation, the VIGS efficiency was evaluated using RT-qPCR analysis with specific primers listed in the Supplementary Table S1. The plants were then agroinfiltrated with the infectious clones of TYLCCNV/TYLCCNB, TYLCCNV/TYLCCNB/TYLCCNA or the mock clone of pBINPLUS as described above.

### 2.7. Statistical Analysis

The data were given as the mean ± standard deviation (SD) of three independent biological replicates. Differences in the mean values were assessed using the SPSS software (v.24.0, SPSS Inc., USA), followed by a one-way ANOVA analysis with the Tukey′s test. Values were considered significantly different at a *p*-value of < 0.05.

## 3. Results

### 3.1. Effects of TYLCCNA on Virus-Infected Symptoms and Accumulation of Viral Genomic DNAs

**Figure 1:**
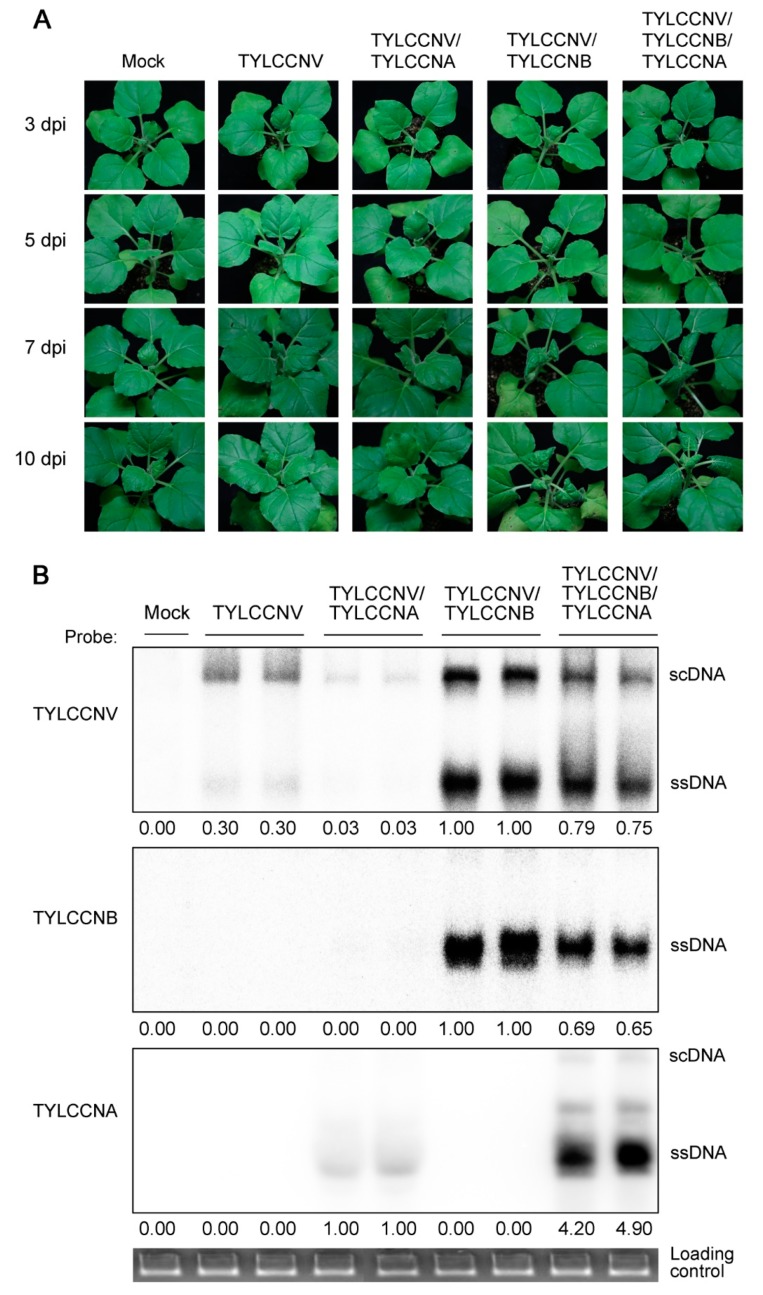
Symptoms and viral DNA accumulation in *N. benthamiana* plants during virus infections. (**A**) Disease symptoms induced by tomato yellow leaf curl China virus (TYLCCNV) alone, TYLCCNV/alphasatellite (TYLCCNA), TYLCCNV/betasatellite (TYLVVNB) or TYLCCNV/TYLCCNB/TYLCCNA in *N. benthamiana* seedlings at 3, 5, 7 and 10 days post-inoculation (dpi). Mock-inoculated with pBINPLUS was used as the control. (**B**) TYLCCNV, TYLCCNB and TYLCCNA genomic DNA levels in *N. benthamiana* seedlings infected with TYLCCNV, TYLCCNV/TYLCCNA, TYLCCNV/TYLVVNB, TYLCCNV/TYLCCNB/TYLCCNA or pBINPLUS at 7 dpi are shown in Figure 1A. Total genomic DNA (approximately 10 μg for each lane) from a mixture of four seedlings was used for the Southern blot. Blots were probed with the coat protein gene sequence of TYLCCNV (top), the full-length sequence of TYLCCNB (middle) and Rep of TYLCCNA (bottom). An ethidium bromide-stained gel shown below the blots provides a DNA-loading control (downmost). The positions of supercoiled (scDNA) and single stranded (ssDNA) forms are indicated, respectively. Quantitative assessment was processed by Quantity One Software (http://www.bio-rad.com).

To investigate the effects of alphasatellite TYLCCNA on the symptoms of virus-infected plants, we examined the time course of symptom development of *N. benthamiana* plants infected by TYLCCNV or/and TYLCCNB with or without TYLCCNA. *N. benthamiana* seedlings at the 5- to 6-leaf stage were agroinoculated with five different groups of infectious clones: TYLCCNV, TYLCCNV/TYLCCNA, TYLCCNV/TYLCCNB, TYLCCNV/TYLCCNB/TYLCCNA and pBINPLUS empty vector (Mock). Consistent with our previous report that *N. benthamiana* seedlings showed no symptoms when agroinoculated with TYLCCNV alone [17], the virus genomic DNA was detected by PCR in all 20 seedlings agroinoculated with TYLCCNV at 7 dpi (Figure 1A and Table 1). When coinfected with TYLCCNV and TYLCCNA, *N. benthamiana* seedlings also had no symptoms (Figure 1A). Consistently, severe symptoms were observed in seedlings upon virus infection in the presence of TYLCCNB. *N. benthamiana* plants coinoculated with TYLCCNV and TYLCCNB were found to develop infection symptoms at 5 dpi, and produced typical infection symptoms, such as vein swelling and leaf curling at 7 dpi (Figure 1A). As expected, coinoculation with TYLCCNV, TYLCCNB and TYLCCNA on *N. benthamiana* seedlings yielded similar infection symptoms as those coinoculated with TYLCCNV and TYLCCNB at 7 and 10 dpi (Figure 1A). Taken together, the results indicate that TYLCCNA has no obvious effects on the infection symptoms induced by TYLCCNV or the TYLCCNV/TYLCCNB complex.

Previous studies have shown that some alphasatellites can decrease the accumulation of their helper viruses and/or betasatellites during viral infections [27,28]. Here, we also examined the accumulation patterns of viral genomic DNA during TYLCCNV/TYLCCNB infection in the presence of TYLCCNA by Southern blot analysis. In the presence of TYLCCNA, the genomic DNA of TYLCCNV and TYLCCNB were both reduced at 7 dpi (Figure 1B). This result indicates that TYLCCNA is capable of reducing the accumulation of viral genomic DNA during TYLCCNV/TYLCCNB infection.

### 3.2. Transcriptome Sequencing of Leaves of N. benthamiana Infected by TYLCCNV/TYLCCNB with or without TYLCCNA

**Figure 2:**
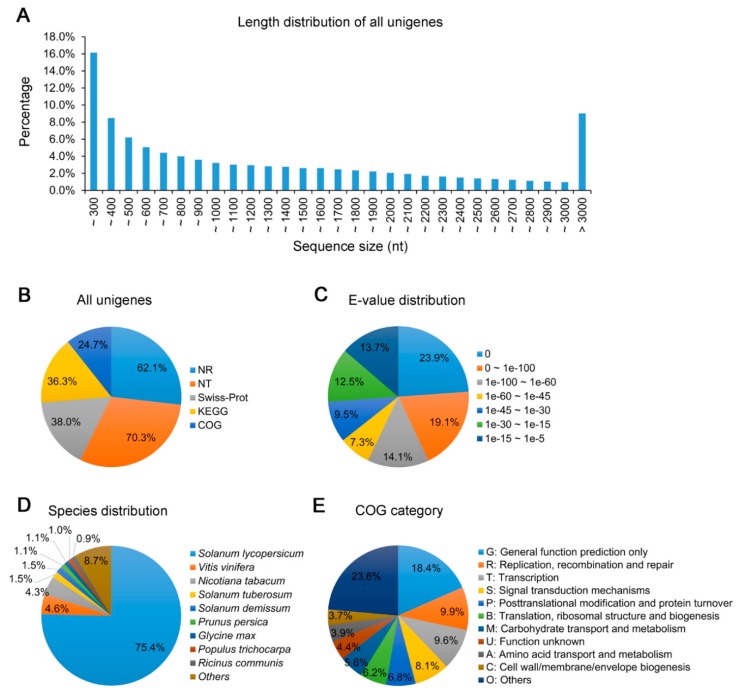
Illumina sequencing of leaves of *N. benthamiana* in response to virus infections. (**A**) The length distribution of all assembled unigenes. (**B**) The number of unigenes annotated by different databases, including NR, NT, Swiss-Prot, Kyoto Encyclopedia of Genes and Genomes (KEGG) and COG. (**C**) E-value distribution of all NR-annotated unigenes. (**D**) Species distribution of all NR-annotated unigenes. (**E**) Category classification of all COG-annotated unigenes.

To explore the DEGs of *N. benthamiana* in response to TYLCCNA during TYLCCNV/TYLCCNB infection, the comprehensive gene expression profiles of leaves of *N. benthamiana* plants infected by TYLCCNV/TYLCCNB with or without TYLCCNA were examined using Illumina sequencing. In this study, for each virus infection, three biological replicates were performed and good correlations were obtained among these three replicates (0.82 < *R*^2^ < 0.99) (Figure S1). More than 96.02% and 91.02% of the clean reads had quality scores at the levels of Q20 and Q30, respectively (Supplementary Table S2). All clean reads obtained from mock or different virus-infected samples were assembled using the Trinity software, resulting in 207,577 unigenes with an average length of 1326 bp and a N50 of 2098 bp. There were 106,143 unigenes (51.1%) with lengths ≤1000 bp, 53,862 unigenes (25.9%) in the range of 1000–2000 bp, 28,829 unigenes (13.9%) in the range of 2000–3000 bp and 18,743 (9.0%) with lengths >3000 bp in our transcriptome datasets (Figure 2A). In total, 151,946 unigenes (73.2%) were annotated using BLASTX algorithm-based software to query against several databases, including NCBI non-redundant protein database (NR), NCBI non-redundant nucleotide database (NT), Swiss-Prot, KEGG and COG. There were 128,986 unigenes (62.1%) matching the protein sequences in the NR database, 146,026 (70.3%) in the NT database, 78,922 (38.0%) in the Swiss-Prot database, 75,439 (36.3%) in the KEGG database and 51,232 (24.7%) in the COG database (Figure 2B). The distribution of the E-values of the annotated unigenes is shown in Figure 2C. Consistently, a mass of *N. benthamiana* unigenes displayed high similarities to the genes annotated in other plant species (Figure 2D). The largest number of *N. benthamiana* homologous genes was identified in *Solanum lycopersicum*, a common vegetable plant. For the COG classification, a total of 51,232 unigenes were divided into 25 COG functional categories. The COG term of ‘general function prediction only’ made up the largest category. Interestingly, 9424 unigenes (18.4%) belonged to the ‘replication, recombination and repair’ category, which contained the second-most of the virus infection responsive genes (Figure 2E).

**Figure 3:**
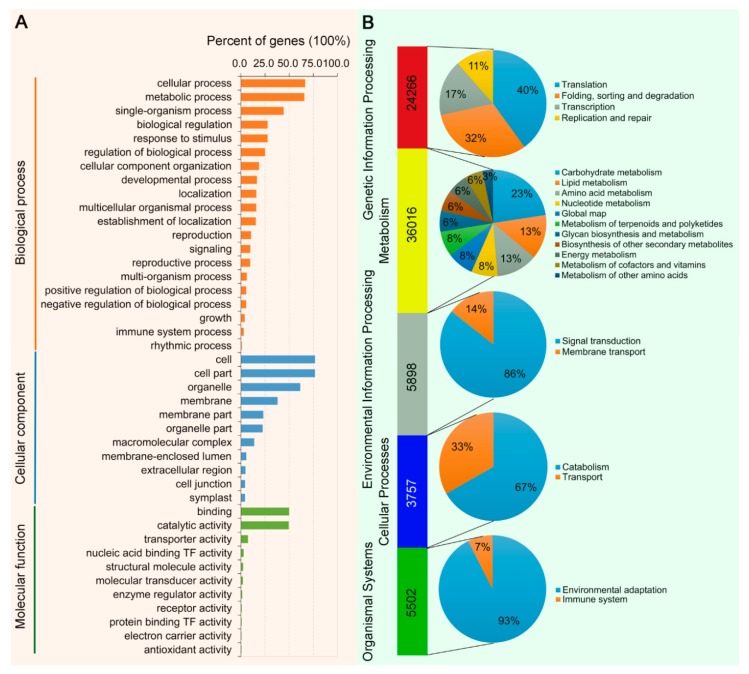
Classification of enriched Gene Ontology (GO) and Kyoto Encyclopedia of Genes and Genomes (KEGG) terms. (**A**) A total of 96,374 unigenes were assigned to different GO terms. (**B**) A total of 75,439 unigenes were assigned to different KEGG terms.

A total of 96,374 unigenes (46.4%) were able to assign to at least one GO term. Within the biological process category, ‘cellular process’, ‘metabolic process’ and ‘single-organism process’ were the three most abundant terms. Within the cellular component category, the three most highly represented terms were ‘cell’, ‘cell part’ and ‘organelle’. The most enriched terms within the molecular function category were ‘binding’, ‘catalytic activity’ and ‘transporter activity’ (Figure 3A). A total of 75,439 unigenes (36.3%) were assigned into various KEGG metabolic/signaling pathways, including pathways related to genetic information processing, metabolism, environmental information processing, cellular process and organismal systems. Interestingly, the most enriched KEGG pathways included those related to metabolic pathways, such as ‘carbohydrate metabolism’ (8128 unigenes), ‘lipid metabolism’ (4861 unigenes), ‘amino acid metabolism’ (4517 unigenes) and ‘nucleotide metabolism’ (2890 unigenes). For genetic information processing, the ‘translation’ term possessed the largest number of unigenes (9708 unigenes). For environmental information processing, the ‘signal transduction’ term contained the largest number of unigenes (5056 unigenes). For organismal systems, the largest number of unigenes (5092 unigenes) was assigned into ‘environmental adaptation’ (Figure 3B).

### 3.3. Screening of DEGs Responding to TYLCCNV/TYLCCNB and TYLCCNV/TYLCCNB/TYLCCNA

**Figure 4:**
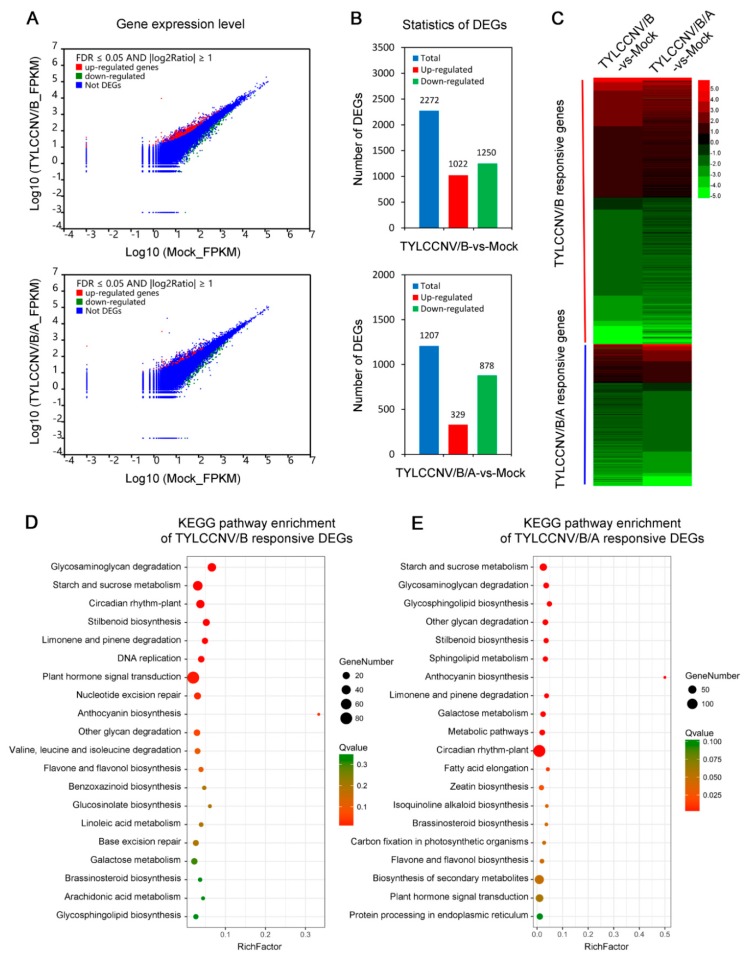
Transcriptional variation of *N. benthamiana* plants in response to TYLCCNV/TYLCCNB (TYLCCNV/B) and TYLCCNV/TYLCCNB/TYLCCNA (TYLCCNV/B/A) infections. (**A**) Differential expression analysis of all differentially expressed genes (DEGs) in leaves of *N. benthamiana* seedlings during TYLCCNV/B or TYLCCNV/B/A infections by volcano plots. (**B**) The numbers of total, up-regulated and down-regulated DEGs in leaves of *N. benthamiana* seedlings during TYLCCNV/B or TYLCCNV/B/A infections. (**C**) Expression profiles of the DEGs between defense responses to TYLCCNV/B and TYLCCNV/B/A infections were shown by a heatmap. The red block indicates the TYLCCNV/B responsive genes and the blue block indicates the TYLCCNV/B/A responsive genes. (**D** and **E**) KEGG analyses of DEGs in response to TYLCCNV/B (**D**) and TYLCCNV/B/A (**E**). The top 20 enriched KEGG terms are shown.

To compare the DEGs between defense responses of *N. benthamiana* seedlings to TYLCCNV/TYLCCNB and TYLCCNV/TYLCCNB/TYLCCNA, FPKM values were used to calculate the read density for each unigene as described in previous research [41]. In the present study, a strict criterion of twofold differences and FDR < 0.05 was used as described in previous research [42,43]. A differential expression analysis of the DEGs between the defense responses of *N. benthamiana* seedlings to TYLCCNV/TYLCCNB and TYLCCNV/TYLCCNB/TYLCCNA was visualized using volcano plots (Figure 4A). A total of 3479 DEGs were identified, which included 2272 TYLCCNV/TYLCCNB responsive genes (Supplementary Table S3) and 1207 TYLCCNV/TYLCCNB/TYLCCNA responsive genes (Supplementary Table S4). The number of TYLCCNV/TYLCCNB responsive genes was nearly twice as much as that of TYLCCNV/TYLCCNB/TYLCCNA. Interestingly, the number of up- and down-regulated genes was nearly equal in response to TYLCCNV/TYLCCNB infection, whilst the down-regulated genes were predominantly following a TYLCCNV/TYLCCNB/TYLCCNA infection (Figure 4B). Furthermore, a hierarchical cluster analysis of these DEGs was performed using a heatmap. As shown in Figure 4C, the DEGs displayed substantial differences in responses of *N. benthamiana* seedlings to TYLCCNV/TYLCCNB and TYLCCNV/TYLCCNB/TYLCCNA infections.

In addition, the DEGs were also mapped to the reference canonical pathways in the KEGG database to identify the differential metabolic pathways between responses of *N. benthamiana* to TYLCCNV/TYLCCNB and TYLCCNV/TYLCCNB/TYLCCNA infections. The DEGs could be significantly enriched into 9 and 18 predicted metabolic pathways by a Q-value < 0.05 after FDR correction, respectively. A big mass of TYLCCNV/TYLCCNB responsive DEGs were mainly enriched in the following KEGG pathways: ‘glycosaminoglycan degradation’, ‘starch and sucrose metabolism’, ‘circadian rhythm-plant’, ‘stilbenoid biosynthesis’, ‘limonene and pinene degradation’ and ‘DNA replication’ (Figure 4D). On the other hand, many TYLCCNV/TYLCCNB/TYLCCNA responsive DEGs were predominantly involved in ‘starch and sucrose metabolism’, ‘glycosaminoglycan degradation’, ‘glycosphingolipid biosynthesis’, ‘other glycan degradation’, ‘stilbenoid biosynthesis’ and ‘sphingolipid metabolism’ KEGG pathways (Figure 4E). Taken together, these results suggest that *N. benthamiana* seedlings may employ different defense strategies against TYLCCNV/TYLCCNB and TYLCCNV/TYLCCNB/TYLCCNA infections.

**Figure 5:**
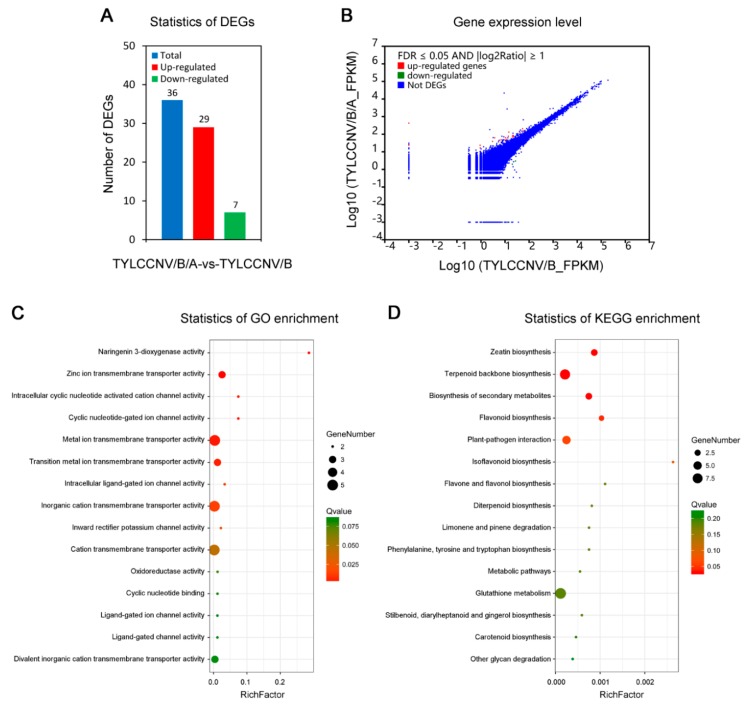
Transcriptome analysis of *N. benthamiana* seedlings in response to TYLCCNA (TYLCCNV/B/A-vs-TYLCCNV/B). (**A**) The numbers of total, up-regulated and down-regulated differentially expressed genes (DEGs) in leaves of *N. benthamiana* seedlings upon TYLCCNV (TYLCCNV/B/A-vs-TYLCCNV/B). (**B**) Volcano plot analysis of DEGs in leaves of *N. benthamiana* seedlings upon TYLCCNV (TYLCCNV/B/A-vs-TYLCCNV/B). (**C**) The GO term enrichment analysis of DEGs in leaves of *N. benthamiana* seedlings upon TYLCCNV (TYLCCNV/B/A-vs-TYLCCNV/B). The top 15 enriched GO terms are shown. (**D**) The KEGG term enrichment analysis of DEGs in leaves of *N. benthamiana* seedlings upon TYLCCNV (TYLCCNV/B/A-vs-TYLCCNV/B). The top 15 enriched KEGG terms are shown.

To identify DEGs responding to TYLCCNA, we next compared the transcriptomes of *N. benthamiana* seedlings infected with TYLCCNV/TYLCCNB/TYLCCNA to those infected with TYLCCNV/TYLCCNB. A total of 36 DEGs, which included 29 up-regulated unigenes and 7 down-regulated unigenes, were identified and analyzed using a criterion of twofold differences and FDR < 0.05 and a volcano plot (Table 2 and Figure 5A,B). Interestingly, the number of up-regulated DEGs responding to TYLCCNA was four times more than that of the down-regulated DEGs (Figure 5A). To further understand host responses to TYLCCNA in *N. benthamiana* seedlings, GO and KEGG enrichment analyses of the 36 DEGs associated with TYLCCNA were performed. Within these DEGs, 10 GO terms of molecular function, such as ‘naringenin 3-dioxygenase activity’, ‘zinc ion transmembrane transporter activity’, ‘intracellular cyclic nucleotide activated cation channel activity’, ‘cyclic nucleotide-gated ion channel activity’ and ‘metal ion transmembrane transporter activity’, were significantly enriched using a threshold value (Q-value < 0.05) (Figure 5C). Moreover, we also mapped these DEGs to the reference canonical pathways in the KEGG database to identify the different metabolic pathways activated in response to TYLCCNA. Twenty-eight of these DEGs could be mapped to 19 predicted metabolic pathways, of which six pathways were highly enriched (*p*-value < 0.05). After FDR correction, four pathways, ‘zeatin biosynthesis’, ‘terpenoid backbone biosynthesis’, ‘biosynthesis of secondary metabolites’ and ‘flavonoid biosynthesis’, were significantly enriched (Q-value < 0.05) (Figure 5D). These results suggest that these molecular functions and metabolic pathways may be associated with the host response to TYLCCNA in *N. benthamiana*.

### 3.4. Validation of Gene Expression by RT-qPCR Analysis

**Figure 6:**
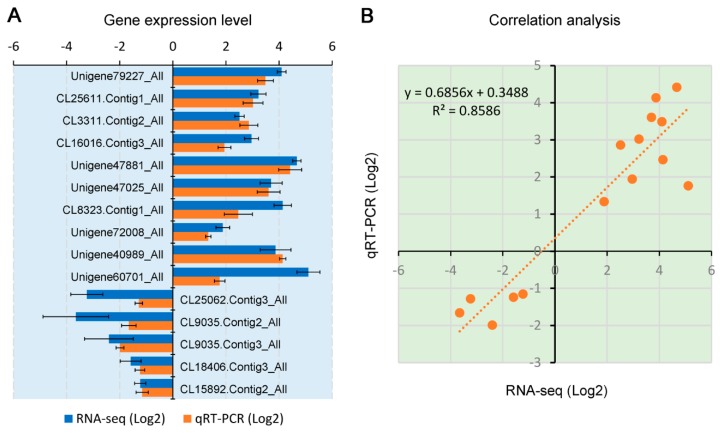
RT-qPCR validation and correlation analysis of gene expression levels between RNA sequencing (RNA-Seq) and RT-qPCR data. (**A**) RT-qPCR analysis of gene expression levels. Fifteen DEGs potentially responding to TYLCCNA were selected and subjected to RT-qPCR analysis using the same RNA as for RNA-Seq. *NbACT2* was used as an internal reference. (**B**) Correlation of gene expression levels between RNA-Seq data and RT-qPCR analysis. Both *x*- and *y*-axes are shown in the Log2 scale.

To verify the results of our RNA-Seq data, 15 DEGs (10 up-regulated and 5 down-regulated) involved in responding to TYLCCNA were randomly selected for RT-qPCR analysis. As shown in Figure 6A, all of these 15 DEGs had similar expression patterns to that were observed by RNA-Seq. The Pearson correlation analysis showed a good correlation (R^2^ = 0.8586) between RNA-Seq data and RT-qPCR results (Figure 6B). These results indicate that the RNA-Seq data accurately imaged the transcriptional alterations induced by virus infections.

### 3.5. Disruption of TYLCCNA Responsive Genes Increases Susceptibility to TYLCCNV/TYLCCNB Infection

**Figure 7:**
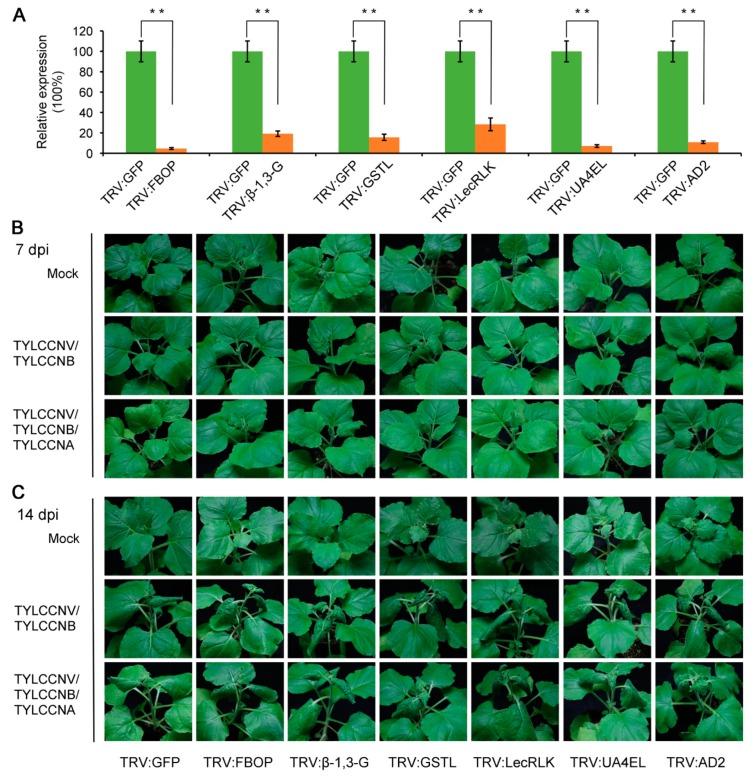
Disruption of the TYLCCNA responsive genes of *N. benthamiana* makes it susceptible to TYLCCNV/TYLCCNB infection. (**A**) Silencing efficiency analysis. The efficiency of VIGS was determined by RT-qPCR at 10 dpi. The values represented relative mRNA levels against control groups (TRV:GFP-infiltrated *N. benthamiana* seedlings), values of which are all set to 100%. The data are shown as means ± standard deviation of three biological replicates. Statistically significant differences in gene expression are indicated with ** (*p* < 0.01). (**B**,**C**) Symptom development in the VIGS-silenced *N. benthamiana* *seedlings* infected with TYLCCNV/TYLCCNB or TYLCCNV/TYLCCNB/TYLCCNA at 7 (**B**) and 14 (**C**) dpi. Mock-inoculated with pBINPLUS was used as the control.

To further investigate the effects of DEGs regulated by TYLCCNA on TYLCCNV/TYLCCNB infection on *N. benthamiana* plants, we selected six TYLCCNA responsive DEGs: *FBOP*, *β-1,3-G*, *GSTL*, *LecRLK*, *UA4EL* and *AD2* based on their molecular functions to examine their potential roles in suppressing virus infection using a VIGS-based approach described in previous research [4]. Approximately 3-week-old *N. benthamiana* plants were agroinfiltrated with the constructed silencing vectors (TRV:FBOP, TRV:β-1,3-G, TRV:GSTL, TRV:LecRLK, TRV:UA4EL and TRV:AD2) or the TRV:GFP control plasmid. The silencing efficiency of VIGS in systemic leaves of the silenced plants was determined by RT-qPCR at 10 dpi. The transcript levels of *FBOP*, *β-1,3-G*, *GSTL*, *LecRLK*, *UA4EL* and *AD2* in *N. benthamiana* seedlings agroinfiltrated with the designed silencing vectors were reduced by approximately 70–90% as compared with those in the TRV:GFP-infiltrated plants (Figure 7A), suggesting that gene silencing was successfully induced by VIGS. Next, the silenced and control plants were inoculated with the infectious clones of TYLCCNV/TYLCCNB, TYLCCNV/TYLCCNB/TYLCCNA or the mock clone of pBINPLUS and monitored for symptom development. The downward leaf curl and vein swelling disease symptoms associated with TYLCCNV/TYLCCNB appeared in *N. benthamiana* seedlings agroinfiltrated by the designed silencing vectors at 7 dpi and became more severe at 14 dpi compared with the TRV:GFP-infiltrated plants (Figure 7B,C). Similar disease symptoms and timing were observed with TYLCCNV/TYLCCNB/TYLCCNA in plants agroinfiltrated by the designed silencing vectors (Figure 7B,C). However, the disease symptoms in *N. benthamiana* plants agroinfiltrated by the designed silencing vectors were slightly weakened in the presence of TYLCCNA (Figure 7C).

**Figure 8:**
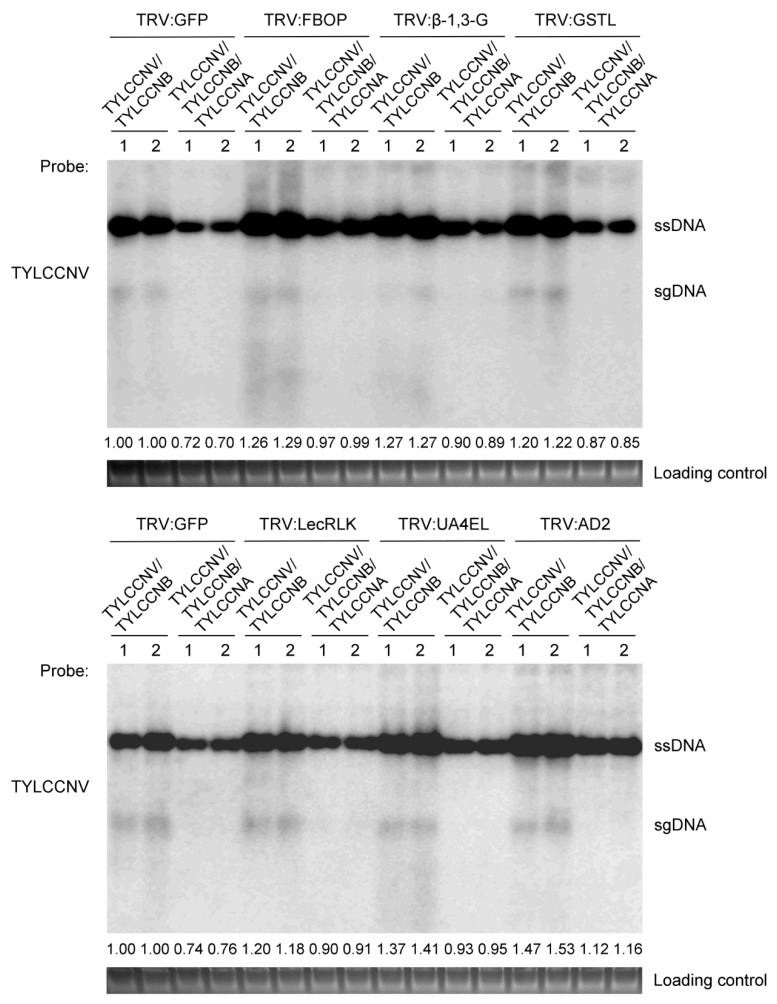
Accumulation levels of TYLCCNV in VIGS-silenced *N. benthamiana* plants infected with TYLCCNV/TYLCCNB or TYLCCNV/TYLCCNB/TYLCCNA at 14 dpi. The accumulation levels of TYLCCNV in VIGS-silenced *N. benthamiana* seedlings were determined by Southern blot shown in Figure 8. Total genomic DNA (approximately 10 μg for each lane) from a mixture of four seedlings was used for Southern blot analysis. Blots were probed with the coat protein gene sequence of TYLCCNV. An ethidium bromide-stained gel shown below the blots provides a DNA-loading control. The positions of single stranded (ssDNA) and subgenomic (sgDNA) forms of TYLCCNV are indicated, respectively. Quantitative assessment was processed by Quantity One Software (http://www.bio-rad.com).

Furthermore, we also determined the viral DNA accumulation in the visible systemic leaves from the TYLCCNV/TYLCCNB- or TYLCCNV/TYLCCNB/TYLCCNA-infected *N. benthamiana* plants at 14 dpi by Southern blot analysis. As shown in Figure 8, for inoculation with TYLCCNV/TYLCCNB, the viral genomic DNA accumulation levels were higher in *N. benthamiana* plants agroinfiltrated with the designed silencing vectors than those in the control seedlings agroinfiltrated with the TRV:GFP. In the presence of TYLCCNA, similar accumulation patterns of viral genomic DNA were obtained in VIGS-silenced and control plants; however, viral DNA accumulation levels were lower than those in plants agroinfiltrated with only TYLCCNV/TYLCCNB (Figure 8). These results indicate that these TYLCCNA responsive DEGs might be disease-resistant genes and play important roles in defense of *N. benthamiana* plants against TYLCCNV/TYLCCNB infection, and that silencing of these genes may enhance the susceptibility of *N. benthamiana* plants against TYLCCNV/TYLCCNB infection. Taken together, these results suggest that TYLCCNA may boost host plant defenses to TYLCCNV/TYLCCNB infection through regulating TYLCCNA responsive genes and thereby moderating the severe damage caused by TYLCCNV/TYLCCNB.

## 4. Discussion

More than 100 alphasatellite sequences have been reported to be associated with begomoviruses or begomovirus/betasatellite complexes since the first alphasatellite was identified with a yellow vein disease in host plants [46]. The genomes of alphasatellites have a predicted hairpin structure and an A-rich region, in which region a range of ∼150- to 200-nt is present with an A content between 46% and 58% [13]. Previously, alphasatellites have only been reported to couple to monopartite begomoviruses which are frequently in association with betasatellites in the Old World [22,27,28,46]. Recently, alphasatellites have also been found in association with bipartite begomoviruses in the New World [29,47,48]. TYLCCNV, which causes a severe yellow leaf curl disease in many crops in China, is a monopartite begomovirus associated with a betasatellite (TYLCCNB) [33,49] and an alphasatellite (TYLCCNA) [22]. Here, our data confirmed that in the presence of TYLCCNA, the accumulation levels of TYLCCNV and TYLCCNB DNAs were greatly reduced during TYLCCNV/TYLCCNB infection, although TYLCCNA had no obvious effects on the viral symptoms of the TYLCCNV/TYLCCNB disease complex (Figure 1). This is in agreement with previous reports showing that some alphasatellites are capable of reducing the accumulation of their helper virus and/or betasatellite DNAs [27,28]. However, the biological functions of alphasatellites during begomovirus/betasatellite infections have not been well studied. Thus, the identification of DEGs responding to TYLCCNA provides the opportunity to determine the biological functions of alphasatellites during the begomovirus/betasatellite infections.

In past years, the availability of transcriptome data from host plants following begomovirus or begomovirus/satellite infections has constantly increased. For instance, data sets from rosette leaves of Arabidopsis plants following cabbage leaf curl virus (CaLCuV) infection having 5365 DEGs were generated by Ascencio-Ibáñez′s group [50], leaves of pepper plants in response to pepper golden mosaic virus (PepGMV) having 309 DEGs were generated by Rivera-Bustamante′s group [51], leaves of tomato plants in response to tomato chlorosis virus (ToCV) and tomato yellow leaf curl virus (TYLCV) having 1568 DEGs were published by Jung′s group [52] and leaves of tobacco plants in response to tobacco curly shoot virus (TbCSV) having 4081 DEGs were published by Qing′s group [53]. In this study, RNA-Seq-based transcriptomic characterization and comparative analysis of TYLCCNV/TYLCCNB- or TYLCCNV/TYLCCNB/TYLCCNA-infected *N. benthamiana* plants with mock-inoculated control seedlings were performed to further understand the biological functions of TYLCCNA during TYLCCNV/TYLCCNB infection. In total, 2272 and 1207 DEGs were screened from TYLCCNV/TYLCCNB-vs-mock and TYLCCNV/TYLCCNB/TYLCCNA-vs-mock, respectively (Figure 4B,C). In the presence of TYLCCNA, the number of DEGs in TYLCCNV/TYLCCNB/TYLCCNA-infected *N. benthamiana* plants was reduced by 1065 compared with that in the plants infected with TYLCCNV/TYLCCNB alone. These results indicated the presence of a large number of transcriptional alterations during begomovirus/satellite infections and that the alphasatellite can affect begomovirus/betasatellite infection at the transcriptome level. Among the top 10 enriched KEGG pathways, ‘glycosaminoglycan degradation’, ‘starch and sucrose metabolism’, ‘circadian rhythm-plant’ and ‘stilbenoid biosynthesis’ were the four most significantly affected pathways in TYLCCNV/TYLCCNB-vs-mock (Figure 4D), suggesting that the disorder of these pathways might contribute to those disease symptoms and viral accumulations caused by TYLCCNV/TYLCCNB in *N. benthamiana* plants. In contrast, the four most significantly affected pathways in response to TYLCCNV/TYLCCNB/TYLCCNA infection were ‘starch and sucrose metabolism’, ‘glycosaminoglycan degradation’, ‘glycosphingolipid biosynthesis’ and ‘other glycan degradation’ (Figure 4E). These results suggest that TYLCCNA might modulate the TYLCCNV/TYLCCNB infection via regulating the ‘starch and sucrose metabolism’ and ‘glycosaminoglycan degradation’ KEGG pathways.

The goal of our transcriptomic analysis was to investigate genes in *N. benthamiana* leaves that were differentially regulated by TYLCCNA and involved in the modulation of TYLCCNV/TYLCCNB infection. We thus compared the transcriptomes of *N. benthamiana* plants infected with TYLCCNV/TYLCCNB/TYLCCNA to those infected with TYLCCNV/TYLCCNB. As a result, a total of 36 DEGs, which included 29 up-regulated unigenes and 7 down-regulated unigenes, were identified in response to TYLCCNA (Figure 5A,B). KEGG enrichment analysis showed that the TYLCCNA responsive DEGs were mainly implicated in the biosynthesis of secondary metabolites and the metabolic pathways (Figure 5C,D). Therefore, TYLCCNA may participate in the resistance of host plants to TYLCCNV/TYLCCNB infection via modulating the biosynthesis and metabolism of related metabolites and upregulating various host genes. This is consistent with previous reports showing that host metabolic pathways are frequently manipulated by begomoviruses or begomovirus/betasatellite disease complexes [53,54].

In previous research, reverse genetic technologies including VIGS have been broadly used in plants to validate the function of specific genes and have become an efficient tool for high-throughput functional genomics [55,56]. The superiorities of TRV-based VIGS over traditional transgenic approaches make it an extremely useful tool for ‘loss-of-function’ gene analysis in *N. benthamiana* plants [57]. Here, a TRV-based VIGS analysis was employed to investigate the biological functions of six TYLCCNA responsive DEGs during TYLCCNV/TYLCCNB infection. Silencing of these genes enhanced the susceptibility of *N. benthamiana* plants to TYLCCNV/TYLCCNB infection and increased the viral DNA accumulation (Figure 7 and Figure 8). These results suggest that TYLCCNA may upregulate the expression of these viral resistance genes to alter viral infections and DNA accumulations during TYLCCNV/TYLCCNB infection.

## 5. Conclusions

In this study, we examined the effects of TYLCCNA on symptom development and viral DNA accumulation following TYLCCNV/TYLCCNB infection. TYLCCNA did not significantly affect viral symptom development but reduced viral DNA accumulation following TYLCCNV/TYLCCNB infection. Screening and bioinformatic analyses of DEGs revealed a substantial transcriptional difference between infections with TYLCCNV/TYLCCNB and TYLCCNV/TYLCCNB/TYLCCNA. The biological functions of six TYLCCNV responsive DEGs were analyzed and verified using a TRV-based VIGS analysis. The RNA-Seq-based transcriptomic data and VIGS analysis shown here provide new information concerning the biological functions of TYLCCNA during TYLCCNV/TYLCCNB infection. These results indicate that the combination of transcriptomics with bioinformatic and VIGS analyses is a useful approach to investigate the biological functions of alphasatellites during begomovirus/betasatellite infections.

## Figures and Tables

**Table 1:** Infectivity and incidence induced by TYLCCNV, TYLCCNV/TYLCCNA, TYLCCNV/TYLCCNB or TYLCCNV/TYLCCNB/TYLCCNA in *N. benthamiana* plants at 7 dpi.

Plant Species	Inoculum ^a^	Infectivity ^b^	Incidence ^c^
*N. benthamiana*	Mock	0/20	0/20
	TYLCCNV	20/20	0/20
	TYLCCNV/TYLCCNA	20/20	0/20
	TYLCCNV/TYLCCNB	20/20	20/20
	TYLCCNV/TYLCCNB/TYLCCNA	20/20	20/20

^a^ Plants inoculated with pBINPLUS was used as the mock control. ^b^ Number of infected plants/number of inoculated plants. ^c^ Number of plants producing symptoms/number of inoculated plants.

**Table 2:** List of differentially expressed genes (DEGs) in response to TYLCCNA in *N. benthamiana* plants.

Gene-ID	Log2 (Ratio) ± SD ^a^	*p*-Value	FDR ^b^	Up-Down Regulation	Annotation
CL16016.Contig3_All	2.963 ± 0.260	6.96 × 10^−12^	1.8 × 10^−7^	up	Beta-1,3-glucanase
CL22155.Contig3_All	2.590 ± 0.447	6.74 × 10^−9^	9.1 × 10^−5^	up	UDP-glycosyltransferase
CL22420.Contig2_All	1.753 ± 0.029	8.83 × 10^−8^	8.5 × 10^−4^	up	Probable 2-oxoglutarate/Fe(II)-dependent dioxygenase
CL25117.Contig26_All	1.404 ± 0.212	6.76 × 10^−7^	5.07 × 10^−3^	up	Pleiotropic drug resistance protein 1
CL2512.Contig3_All	2.291 ± 0.314	1.19 × 10^−9^	2.2 × 10^−5^	up	DNA-binding protein 3
CL25611.Contig1_All	3.222 ± 0.286	1.39 × 10^−18^	1.4 × 10^−13^	up	Cyclic nucleotide-gated ion channel 1-like
CL2689.Contig5_All	1.970 ± 0.324	1.06 × 10^−8^	1.3 × 10^−4^	up	WRKY transcription factor 6
CL3311.Contig2_All	2.511 ± 0.175	1.03 × 10^−14^	5.2 × 10^−10^	up	Flavonoid biosynthesis oxidoreductase protein
CL4894.Contig1_All	2.145 ± 0.249	1.49 × 10^−9^	2.5 × 10^−5^	up	Zinc finger CCCH domain-containing protein 2-like
CL6208.Contig1_All	1.583 ± 0.221	2.12 × 10^−6^	1.43 × 10^−2^	up	Receptor-like protein kinase
CL6421.Contig3_All	2.214 ± 0.536	3.31 × 10^−6^	2.09 × 10^−2^	up	Cucumber peeling cupredoxin-like
CL8323.Contig1_All	4.138 ± 0.328	8.51 × 10^−8^	8.5 × 10^−4^	up	Lectin-domain receptor-like kinase
Unigene13300_All	1.627 ± 0.290	2.64 × 10^−8^	2.8 × 10^−4^	up	Putative disease resistance protein
Unigene23199_All	1.979 ± 0.044	1.07 × 10^−17^	7.2 × 10^−13^	up	Retrovirus-related Pol polyprotein
Unigene23569_All	1.878 ± 0.203	1.95 × 10^−7^	1.71 × 10^−3^	up	Copia-type pol polyprotein
Unigene23982_All	1.479 ± 0.254	7.24 × 10^−6^	4.07 × 10^−2^	up	Putative polyprotein
Unigene2441_All	2.759 ± 0.302	6.62 × 10^−6^	3.83 × 10^−2^	up	Cytochrome P450
Unigene28354_All	1.823 ± 0.140	3.77 × 10^−9^	5.8 × 10^−5^	up	Putative gag protein
Unigene30685_All	1.627 ± 0.264	3.58 × 10^−7^	3.02 × 10^−3^	up	Cyclic nucleotide-gated ion channel 1-like
Unigene40989_All	3.869 ± 0.578	1.62 × 10^−6^	1.13 × 10^−2^	up	Arogenate dehydrogenase 2
Unigene46047_All	2.831 ± 0.298	1.16 × 10^−12^	3.9 × 10^−8^	up	Ethylene-responsive transcription factor 1B-like
Unigene47025_All	3.700 ± 0.417	3.38 × 10^−10^	6.9 × 10^−6^	up	GDSL esterase/lipase 2-like
Unigene47881_All	4.669 ± 0.157	3.19 × 10^−11^	7.2 × 10^−7^	up	Glutathione S-transferase-like
Unigene60701_All	5.105 ± 0.434	3.07 × 10^−6^	2.01 × 10^−2^	up	Glutaredoxin-C9-like
Unigene72008_All	1.880 ± 0.258	4.38 × 10^−7^	3.55 × 10^−3^	up	UDP-arabinose 4-epimerase 1-like
Unigene79227_All	4.098 ± 0.163	3.41 × 10^−22^	6.9 × 10^−17^	up	Aspartic proteinase nepenthesin-2-like
CL15892.Contig2_All	−1.229 ± 0.210	8.02 × 10^−6^	4.39 × 10^−2^	down	Zinc transporter 4
CL18406.Contig3_All	−1.589 ± 0.393	6.45 × 10^−6^	3.83 × 10^−2^	down	Probable carboxylesterase 15-like
CL25062.Contig3_All	−3.238 ± 0.604	8.07 × 10^−9^	1.0 × 10^−4^	down	Prolyl endopeptidase-like
CL9035.Contig2_All	−3.655 ± 1.234	1.01 × 10^−7^	9.3 × 10^−4^	down	Zinc transporter 1-like
CL9035.Contig3_All	−2.406 ± 0.918	4.75 × 10^−7^	3.7 × 10^−3^	down	Zinc transporter 8
Unigene6461_All	12.250 ± 0.227	2.19 × 10^−14^	8.9 × 10^−10^	up	Uncharacterized
Unigene75053_All	14.336 ± 1.124	4.41 × 10^−12^	1.3 × 10^−7^	up	Uncharacterized
Unigene78447_All	1.329 ± 0.207	1.16 × 10^−8^	1.3 × 10^−4^	up	Uncharacterized
CL25997.Contig7_All	−8.840 ± 0.000	4.03 × 10^−9^	5.8 × 10^−5^	down	Uncharacterized
CL4777.Contig2_All	−6.532 ± 4.144	3.65 × 10^−6^	2.24 × 10^−2^	down	Uncharacterized

^a^ The data representsmean ± SD (standard deviation) of three independent biological replicates. ^b^ *p*-values were corrected using the Benjamini–Hochberg (BH) method.

## Supplementary Materials

The following are available online at https://www.mdpi.com/1999-4915/11/5/442/s1, Table S1: List of primers used in this study; Table S2: The detailed information of the obtained reads; Table S3: List of DEGs in response to TYLCCNA/TYLCCNB in *N. benthamiana* plants; Table S4: List of DEGs in response to TYLCCNA/TYLCCNB/TYLCCNA in *N. benthamiana* plants; Figure S1: Pearson correlation between the tested samples in the transcriptome sequencing analysis.
