# Supplementary figures and images for: Identification and Analysis of Potential Genes Regulated by an Alphasatellite (TYLCCNA) that Contribute to Host Resistance against Tomato Yellow Leaf Curl China Virus and Its Betasatellite (TYLCCNV/TYLCCNB) Infection in Nicotiana benthamiana
Source: Viruses. 2019 May 15;11(5):442. doi: 10.3390/v11050442 (PMC6563268; doi:10.3390/v11050442)

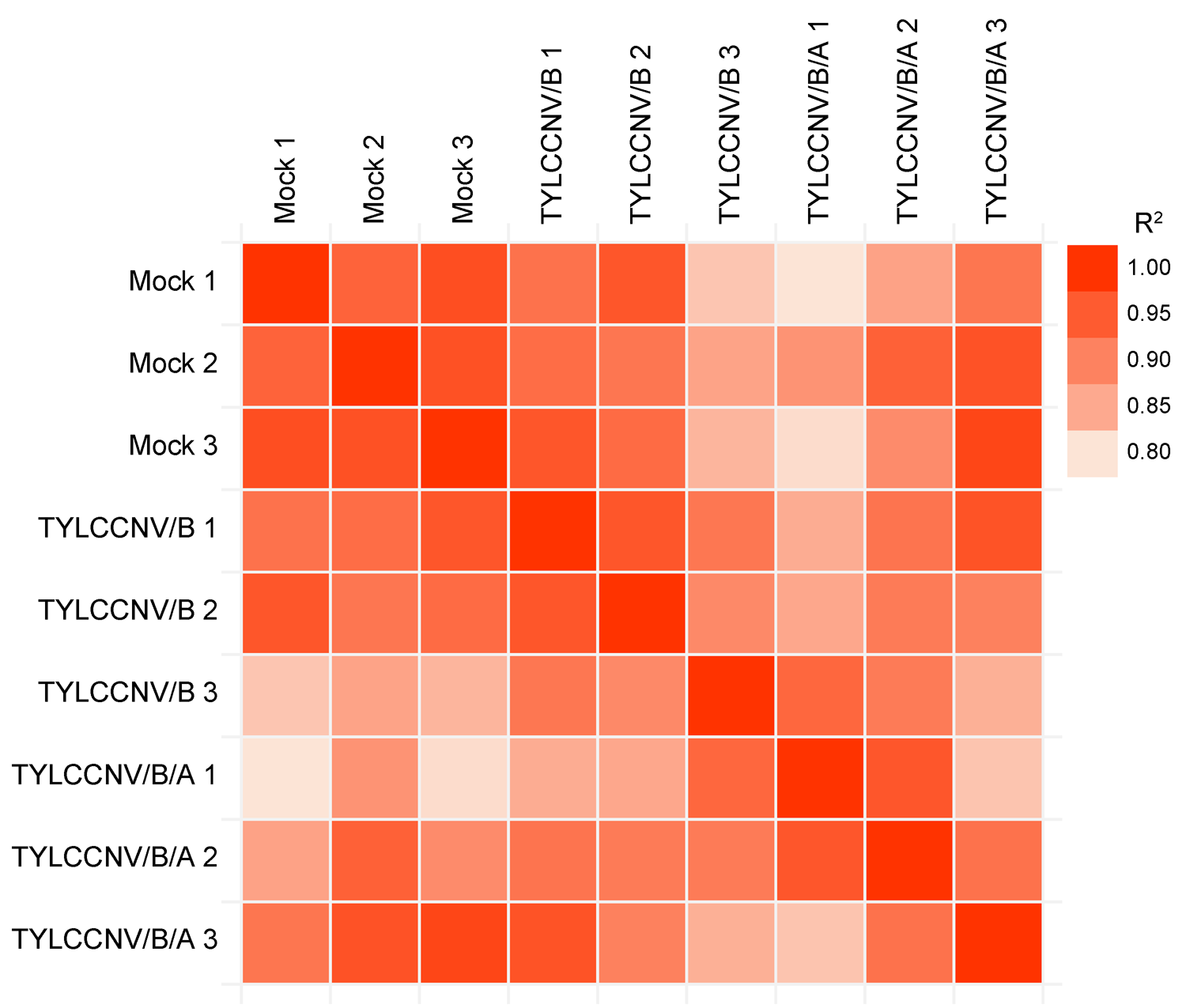

Supplement: Supplementary file 1 [file viruses-11-00442-s001.zip › Figure S1.tif]
